# The genome assembly and annotation of the cricket *Gryllus longicercus*

**DOI:** 10.1038/s41597-024-03554-z

**Published:** 2024-06-28

**Authors:** Szymon Szrajer, David Gray, Guillem Ylla

**Affiliations:** 1 Laboratory of Bioinformatics and Genome Biology, Faculty of Biochemistry, Biophysics and Biotechnology, Jagiellonian University, Kraków, 30-387 Poland; 2 Department of Biology, California State University Northridge, Northridge, CA 91330-8303 USA

**Keywords:** Genome, Genome informatics

## Abstract

The order Orthoptera includes insects such as grasshoppers, katydids, and crickets, among which there are important species for ecosystem stability and pollination, as well as research organisms in different fields such as neurobiology, ecology, and evolution. Crickets, with more than 2,400 described species, are emerging as novel model research organisms, for their diversity, worldwide distribution, regeneration capacity, and their characteristic acoustic communication. Here we report the assembly and annotation of the first New World cricket, that of *Gryllus longicercus* Weissman & Gray 2019. The genome assembly, generated by combining 44.54 Gb of long reads from PacBio and 120.44 Gb of short Illumina reads, has a length of 1.85 Gb. The genome annotation yielded 19,715 transcripts from 14,789 gene models.

## Background & Summary

*Gryllus longicercus*, owns its name to its characteristically long cerci, having the longest known cerci in the genus^1^. It is one of the circa 35 named North American *Gryllus* species, and lives in rocky landscapes of the Sonoran and Chihuahuan deserts of the western USA and Mexico^1,2^.

Crickets, and especially species from the genus *Gryllus*, have been widely used as experimental organisms in evolutionary biology, development, regeneration, neurophysiology, and behavior^3,4^. Examples of such are the two-spotted field cricket *Gryllus bimaculatus* and the *Gryllus campestris*, both belonging to Old World crickets.

Reference genome assemblies and annotations are important tools for biological research, and most especially for evolutionary biology^4^, taxonomy, and conservation of biodiversity^5^ studies. At the time of writing, there are six cricket species (Gryllidae family) that have a publicly available genome assembly, these are: *Laupala kohalensis*^6,7^ (2017), *Teleogryllus oceanicus*^8^ (2019), *Teleogryllus occipitalis*^9,10^ (2020), *Acheta domesticus*^11–13^ (2020, 2023), *Gryllus bimaculatus*^14,15^ (2021), and *Apteronemobius asahinai*^16,17^ (2021).

Here, we present the assembly and annotation of the first New World cricket genome, that of *Gryllus longicercus*. The genome assembly contains 1.85 Gb, spread across 1,571 scaffolds. Our annotation identified 19,715 transcripts belonging to 14,789 gene models.

## Methods

### Sample collection, DNA and RNA extraction, library preparation and sequencing

To obtain genetic material to construct the reference genome, we collected a single adult male of *G. longicercus* in Yuma County, Arizona, USA. Directly following the collection, we dissected out the gut and submerged the sample in 100% ethanol. The sample was sent on dry ice to a sequencing facility (Novogene Co. Inc.). There, insect tissues excluding gut were rinsed twice with PBS to remove trace amounts of ethanol. The tissues were then dissected and placed in 1.5 ml tubes containing 800 µl DNA/RNA shield and incubated for an hour before being further mechanically broken down with pestles. To induce digestion 400 µl of Solid Tissue Buffer and 20 µl of Proteinase K were added per tube and mixed. After incubation with occasional inversion of the tubes at 55 °C in a water bath for 3 hours the remaining tissue fragments were broken down with a squisher. The lysate was then spun down and the supernatant was purified using Quick-DNA™ HMW MagBead Kit with all steps performed on a rotator. Elutes from each tube were pooled together to produce the final purified HMW DNA. Purified genomic DNA served as a template for library construction for two sequencing platforms. The library sequenced with PacBio platform was prepared following the SMRTbell™ Express Template Prep Kit 2.0 and the one sequenced on Illumina platform was constructed following the NEB Ultra II kit.

The RNA material used to create the RNA-Seq libraries, necessary for the genome annotation, was obtained from virgin adult males and females 7–10 days after molting, from the same source population as the reference genome individual. For each sex, we sequenced nine samples, three from the brain, three from the thoracic ganglia, and three from the thoracic plus abdominal ganglia. Crickets were cold anesthetized and then dissected submerged under RnaLater® and sent to Azenta (Azenta, Inc.). Total RNA was extracted from tissues in RnaLater® samples using Qiagen RNeasy Plus Universal Micro Kit, and libraries were prepared with NEBNext Ultra II RNA Library Prep Kit. Libraries were sequenced using a 2x150bp Paired End configuration. Raw sequence data was converted into fastq files and demultiplexed using Illumina’s bcl2fastq (v.2.17) (RRID:SCR_015058), one mismatch was allowed for index sequence identification.

### De novo genome assembly

**Fig. 1:**
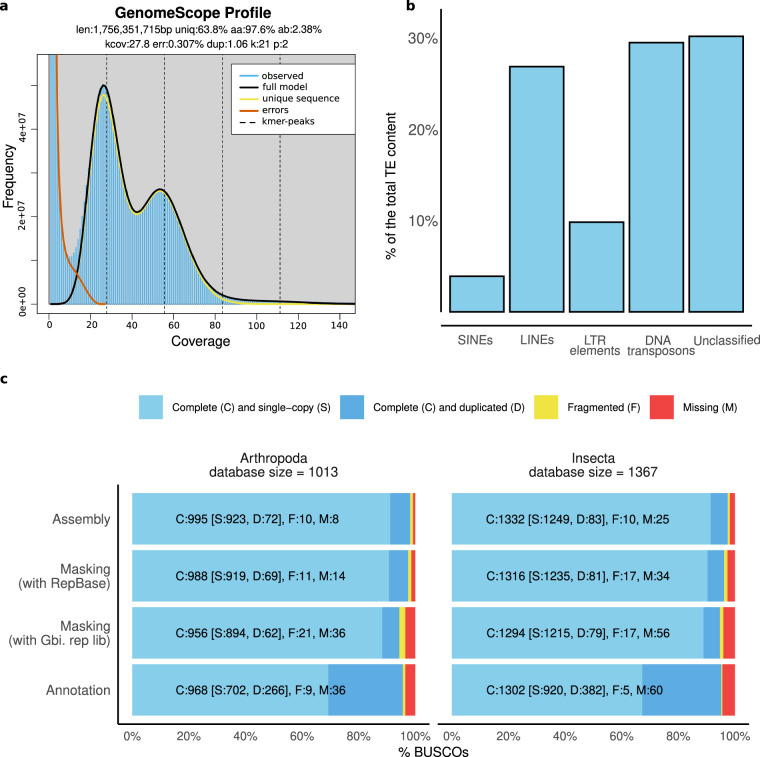
Overview of *G. longicercus* genome sequencing, assembly, and annotation. (**a**) GenomeScope k-mer distribution which estimated the genome length to be 1,76 Gb. (**b**) Proportion of the genome identified as interspersed repeats classified as SINEs, LINEs, LTR elements, DNA transposons, and unclassified. (**c**) BUSCO assessment results of the whole genome assembly, the assembly masked with Repbase library, the assembly masked with *G. bimaculatus* repeat library, and all transcripts obtained during the annotation.

The sequencing yielded a total of 164.98 Gb of raw sequencing data, 120.44 Gb of PE150 from Illumina and 44.54 Gb from PacBio platform with an average read length of 19,924 bp. The data was assembled following the PacBio assembly pipeline using the Hifiasm^18,19^ assembler. With Meryl (v.1.3)^20^ Galaxy (v.6)^21–23^ and using the 120.44 Gb of Illumina reads we performed a k-mer (k = 21) frequency estimation. Based on k-mer histogram we profiled the genome in GenomeScope2 (v.2)^24^ Galaxy (v.2), which resulted in size estimation of 1.76 Gb (Fig. 1a). Based on this estimate, the volume of data generated equals to a coverage of 68.43X and 25.31X for Illumina and PacBio respectively.

The initial assembly obtained had a length of 1.86 Gb (1,858,024,830 nucleotides). To curate this assembly, we first put it through Purge Haplotigs (v1.1.3)^25^ which identified 4 repeated scaffolds, 11 haplotig scaffolds, and 64 junk. These 79 scaffolds were removed from the assembly. To check whether the assembly could contain mitochondrial DNA, we aligned all scaffolds against the three available cricket mitochondrial genomes^26–28^ using Minimap2 (v.2.28)^29^. Minimap2 identified eight scaffold regions with an alignment longer than 10,000 nucleotides and an identity higher than 42% to the cricket mitochondrial genomes. We build a phylogenetic tree of these eight scaffold regions together with the mitochondrial genomes of *G. bimaculatus*^26^, *Gryllus lineaticeps*^27^, *Gryllus veletis*^28^, *Aedes aegypti*^30^, *Blattella germanica*^31^, and *Drosophila melanogaster*^32^ with MUSCLE (v.5.1)^33^ and FastTree 2 (v.2.1.11)^34^ (Supplementary Figure S1). Based on the tree, four regions were identified to be mitochondrial. The scaffolds containing the mitochondrial DNA were split in two, excluding the mitochondrial region from the assembly. To test for possible bacterial contamination, we used BUSCO (v5.5.0)^35,36^ with Bacteria gene set and explored whether any scaffold contained putative bacterial genes. Twenty scaffolds had a single complete possible bacterial BUSCO. A single putative bacterial gene in a scaffold, was considered to be not strong enough evidence as to consider that the scaffold was contaminated, and hence, no scaffolds were removed at this step.

After filtering out 79 scaffolds and removing the mitochondrial DNA, the genome assembly of *G. longicercus* expands 1,850,503,451 nucleotides and comprises 1,571 scaffolds with N90 of 743 and L90 of 612.44Kb. To test its completeness, we used BUSCO, which resulted in a BUSCO score of 98.2%, with 91.1% in single-copy and 7.1% duplicated (S:91.1%, D:7.1%) at Arthropoda and 97.5% (S:91.4%, D:6.1%) at Insecta level (Fig. 1c).

### Repeat masking

Before performing the genome annotation, we proceeded to mask the repetitive content. First, we attempted to mask the genome based on RepBase-derived library (v.20181026)^37^ and using RepeatMasker (v.4.1.4)^38^, which led to a 10.45% of masked genome **(**Supplementary Table S1) and a BUSCO score of 97.5% (S:90.7%, D:6.8%) at Arthropoda and 96.2% (S:90.3%, D:5.9%) at Insecta level on the masked genome (Fig. 1c). Given that these repetitive content values were lower than expected (33–35%) based on other cricket genomes^14^ we attempted to mask the *G. longicercus* genome using the repetitive content library that we previously generated for *G. bimaculatu*s^14^, also belonging to the *Gryllus* genus. This second attempt yielded masking at a level of 51.13% of genome, and BUSCO scores of 94.4% (S:88.3%, D:6.1%) at Arthropoda and 94.7% (S:88.9%, D:5.8%) at Insecta level. Given that this second strategy increased the percentage of masking to the expected amounts without a significant impact on the BUSCO scores, we proceeded to annotate the genome masked with this second strategy.

The most abundant type of repetitive content in *G. longicercus* are DNA transposons at 12.55% of the genome followed by LINEs at 11.33%. The interspersed repeat content totals 42.55% of the genome while repetitive content overall amounts to 51.13% (Fig. 1b and Supplementary Table S2).

### Gene prediction and structural annotation

For structural gene annotation we used BRAKER3 (v.3.0.3)^39–43^ with the protein and RNA-Seq evidence mode (pipeline C)^44–51^. To construct a database of protein evidence we used a foundation of 2.1 Gb Arthropoda proteins from OrthoDB (v.11)^52^ and supplemented it with 26,647 proteins from other Gryllidae species deposited in the NCBI. These were obtained by searching for *Gryllus* Taxonomy ID and choosing nonduplicate records. As for RNA-Seq evidence, we used 204,764,789 RNA-Seq reads from 18 transcriptomes generated in this study. Raw reads were adapter trimmed using Trim Galore! (v.0.6.10)^53^, aligned to the genome with HISAT2 (v.2.2.1)^54^, and the.sam files converted to.bam with Samtools (v.1.17)^45^. With all this data, BRAKER3 predicted 14,789 gene models with a total of 19,715 transcripts. The BUSCO scores of the annotated transcriptome (including splicing isoforms) was 95.6% (S:69.3%, D:26.3%) for Arthropoda and 95.2% for Insecta (S:67.3%, D:27.9%) (Fig. 1c).

### Functional annotation

We proceed with the functional annotation of genes through homology-based approach with series of BlastP (v.2.9.0+)^55,56^ queries on their longest transcript per gene. First, transcripts were queried against all 9,690 Swiss-Prot proteins of insects^57^, and assigned as homologous the best BlastP hit with a significance threshold (e-value < 1e-6). Those transcripts with no significant hit, were queried against all 5,683,201 insect proteins from TrEMBL with the same significance threshold. Those transcripts without significant hit, were queried once more against the entire Swissprot database of 569,793 proteins. Search against Swissprot Insecta annotated 7,544 of total transcripts. The second search has resulted in a database match for another 5,241 and the third one allowed us to obtain a match for additional 7. In total, longest transcripts of 12,792 genes (86.50%) have been functionally annotated with homologous UniProt sequences.

We further expanded genome annotation by executing InterProScan (v.5.63–95.0)^58,59^ resulting with InterPro ID match for 10,704 (72.38%) of longest transcripts per gene as well as at least one Pfam domain match for 10,939 (73.97%) and GO term annotation for 6,614 (44.72%). In total 12,864 (86.98%) of genes have at least one of the four functional annotations of their longest transcripts (Table 1).

**Table 1:** *G. longicercus* functional annotation summary, indicating the number of annotated protein-coding genes, and the percentage of them with significant BlastP hit, and with InterPro ID, Pfam, or GO terms assigned to them.

Annotated protein-coding genes	14,789
With significant BlastP hit	86.50%
With InterPro ID	72.38%
With Pfam domain	73.97%
With GO term	44.72%
With any	86.98%

### Gryllidae assembly and annotation comparison

**Fig. 2:**
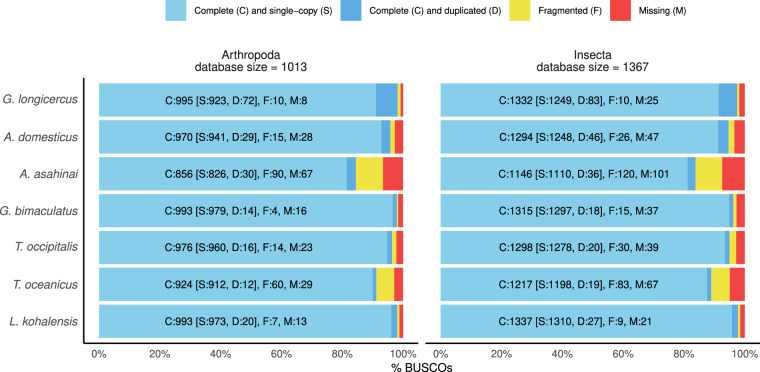
Completeness of cricket genome assemblies. BUSCO assessment results at Arthropoda and Insecta levels of the genome assembly of the six publicly available cricket genome assemblies and *G. longicercus*.

To put the *G. longicercus* genome in the context of the currently available cricket genomes, we obtained the assembly summary statistics of the six existing assemblies (*Laupala kohalensis*^7^*, Teleogryllus oceanicus*^8^, *Teleogryllus occipitalis*^10^, *Gryllus bimaculatus*^15^, *Apteronemobius asahinai*^17^ and *Acheta domesticus*^13^) with BBtools (v.39.01)^60^ (Table 2). The size of the *G. longicercus* genome herein obtained (1.85 Gb) is within the range of the other available cricket genomes (1.6 to 2.3 Gb). Furthermore, in terms of assembly completeness, using as proxy the BUSCO scores, shows the highest completeness scores in Arthropoda and second highest in Insecta (Fig. 2). In terms of contiguousness, the *G. longicercus* assembly displays the third lowest N90, third highest L90 and the lowest number of total scaffolds. The *A. domesticus* genome^12^ is currently the only publicly available cricket genome assembled at a chromosome level with the 11 chromosomes containing more than the 90% of the genome. After that genome, the genome assembly of *G. bimaculatus* and the herein presented assembly of *G. longicercus* are the most complete and contiguous cricket genomes.

**Table 2:** Comparison of the assembly statistics of the currently available cricket genomes.

	*L. kohalensis*	*T. oceanicus*	*T. occipitalis*	*G. bimaculatus*	*A. asahinai*	*A. domesticus*	*G. longicercus*
Size	1.595 GB	1.934 GB	2.045 GB	1.658 GB	1.676 GB	2.346 GB	1.851 GB
Number of scaffolds	148,784	19,866	197,871	47,877	151,060	9,907	1,571
N90	3,483	9,698	82,098	307	73,474	11	743
L90	0.068 MB	0.003 MB	0.048 MB	1.043 MB	0.004 MB	105.625 MB	0.612 MB
% of Genome in scaffolds >50 KB:	91.37%	89.55%	53.88%	95.59%	26.93%	93.30%	99.89%

**Fig. 3:**
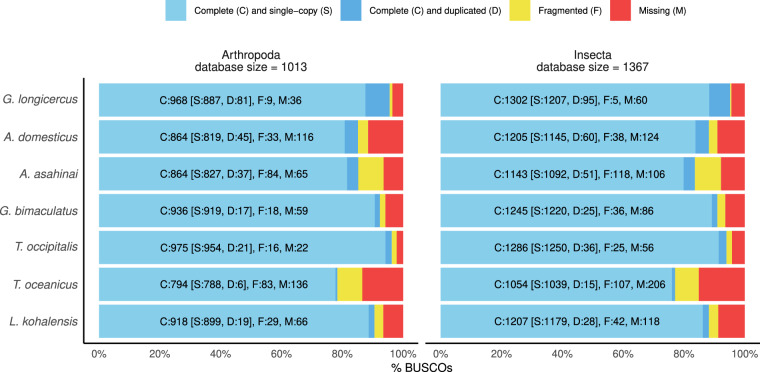
Completeness of the cricket genome annotations. BUSCO assessment results at Arthropoda and Insecta levels of the longest transcript per gene of the six publicly available cricket genome assemblies and *G. longicercus*.

We also compared the genome annotations based on the information from genome annotations files (GFF3 or GTF), except for *A. domesticus*, for which we could not find such file and we used the published fasta files with the sequences of the annotated transcripts. Based on these files we observed that the number of annotated genes in our cricket (14,789) is in the same order of magnitude as other crickets (12,767 to 29,304) (Table 3). Notably, the set of longest transcripts per gene of *G. longicercus* displays the longest average CDS length, second biggest number of exons per gene and third lowest number of single-exon genes out of the compared crickets (Table 3). The annotation is also in the upper bound of the quality indicators compared to that of the other crickets, such as BUSCO completeness score of 95.6% at Arthropoda (ranging from 78.4% to 96.3% in the other crickets) and 95.2% at Insecta level (77.1% to 95.2%) (Fig. 3). These values suggest that our *G. longicercus* annotation might be among the most complete cricket genome annotations.

**Table 3:** Comparison of the genome annotation statistics of the available annotated cricket genomes.

	*L. kohalensis*	*T. oceanicus*	*T. occipitalis*	*G. bimaculatus*	*A. asahinai*	*A. domesticus*	*G. longicercus*
Number of genes	12,767	19,157	20,735	17,871	28,285	29,304	14,789
Average CDS length (bp)	1,358	1,142	1,359	1,206	999	NA	1,397
Single exon genes	1,144	1,266	5,449	3,180	6,668	NA	2,627
Average exon per gene	6.75	5.21	5.54	4.28	4.33	NA	6.37

## Data Records

All the genomic data used in this study can be accessed through NCBI. The initial genome assembly and annotation are available under GenBank identifier JAZDUA000000000.1^61^. The genome assembly and annotations after removing haplotigs and mitochondrial contaminations, based on which the herein analysis have been performed, are available in FigShare^62^. The raw reads from Illumina and PacBio, as well as the transcriptomics datasets used for the annotation, are accessible under the Sequence Read Archive identifier SRP485514^63^.

## Technical Validation

PacBio Genomic DNA samples were quantitated with Qubit® Fluorometer and their integrity and purity was tested with agarose gel electrophoresis. Later, PacBio libraries were once again checked with Qubit® for quantification and bioanalyzer for size distribution selection. For Illumina Genomic DNA samples, Agilent 5400 was used for quantitation as well as the testing of integrity and purity. Libraries from this platform were checked with Qubit® and real-time PCR for quantification and bioanalyzer for size distribution selection. Similarly, the RNA-Seq libraries quality was analyzed on the Agilent TapeStation and quantified using Qubit® Fluorometer. The sequencing libraries were sequenced on a lane of Illumina HiSeq flow cell.

In terms of technical validations on the obtained original assembly, as described, we check for scaffolds that could correspond to haplotigs or that were repeated. We also checked for possible mitochondrial and bacterial contaminations. Handling those we removed ~0.5% of the original assembly. We used BUSCO scores and descriptive statistics as quality indicators for the assembly, for the genome masking, and for the gene annotation. Additionally, we calculated the same indicators on the other cricket genomes published in recent years, which showed that our genome assembly and annotation align with current standards. Overall, the herein reported genome assembly and annotations show a high degree of completeness.

**Fig. 4:**
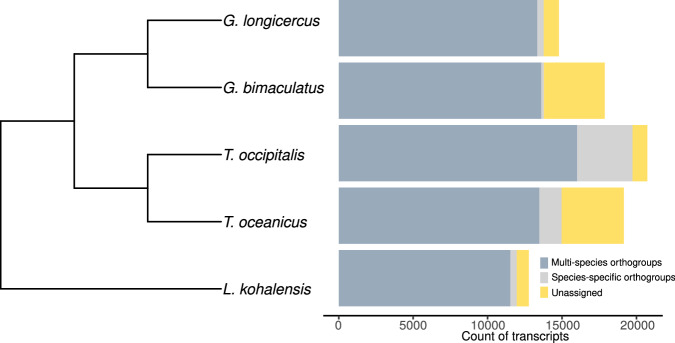
Orthology of the *G. longicercus* annotated genes in crickets *G. bimaculatus*, *T. occipitalis*, *T. oceanicus*, and *L. kohalensis*. The species tree was inferred with Orthofinder with the longest transcripts per gene, with the barplot showing the numbers of transcripts assigned to multi-species orthogroups, species-specific orthogroups, and not assigned to any orthogroup.

To examine the quality of the herein-predicted genes we used OrthoFinder (v. 2.5.5)^64–66^ on the amino acid sequences of the longest annotated transcript per gene on four other species of crickets with available annotations of genes and transcript isoforms (Fig. 4). This analysis returned the expected phylogenetic relationship between cricket species and showed that among the 14,789 genes annotated in *G. longicercus*, 13,332 (90.15%) were assigned multi-species orthogroups, indicating the presence of orthologs in other crickets, and only 1,457 genes (9.85%) were found species-specific. This low number of annotated genes in *G. longicercus* without ortholog in other crickets also supports the robustness of the gene annotations.

### Supplementary information

Supplementary Information

## Data Availability

The code used for the genome annotation and the analysis is available on GitHub (https://github.com/ylla-lab/Gryllus_longicercus) and was deposited in Zenodo with the following doi (10.5281/zenodo.10628205).
