# Supplementary material for: The genome assembly and annotation of the cricket Gryllus longicercus
Source: Sci Data. 2024 Jun 28;11:708. doi: 10.1038/s41597-024-03554-z (PMC11213874; doi:10.1038/s41597-024-03554-z)
Supplement: Supplementary file 1 — Supplementary Information [file 41597_2024_3554_MOESM1_ESM.pdf]

Supplementary Information for:

The genome assembly and annotation of the cricket *Gryllus longicercus*

Szymon Szrajer[1], David Gray[2*],Guillem Ylla[1*]

[1] Laboratory of Bioinformatics and Genome Biology, Faculty of Biochemistry, Biophysics and Biotechnology, Jagiellonian University, Kraków 30-387, Poland
[2] Department of Biology, California State University Northridge, Northridge, CA 91330-8303, USA

* Correspondence to: guillem.ylla@uj.edu.pl, dave.gray@csun.edu

Table of contents:

Supplementary Figure S1: 2
Supplementary Table S1: 3
Supplementary Table S2: 4

Supplementary Information

Supplementary Figure S1: Phylogenetic tree build with MUSCLE (v.5.1) and FastTree 2 (v.2.1.11) with the eight regions with putative mitochondrial DNA contamination and the mitochondrial genomes of *Gryllus bimaculatus*, *Gryllus lineaticeps*, *Gryllus veletis*, *Aedes aegypti*, *Blattella germanica*, and *Drosophila melanogaster.* Based on this tree, the four regions marked with a red asterisk were considered to be mitochondrial DNA contaminations and excluded from the assembly.

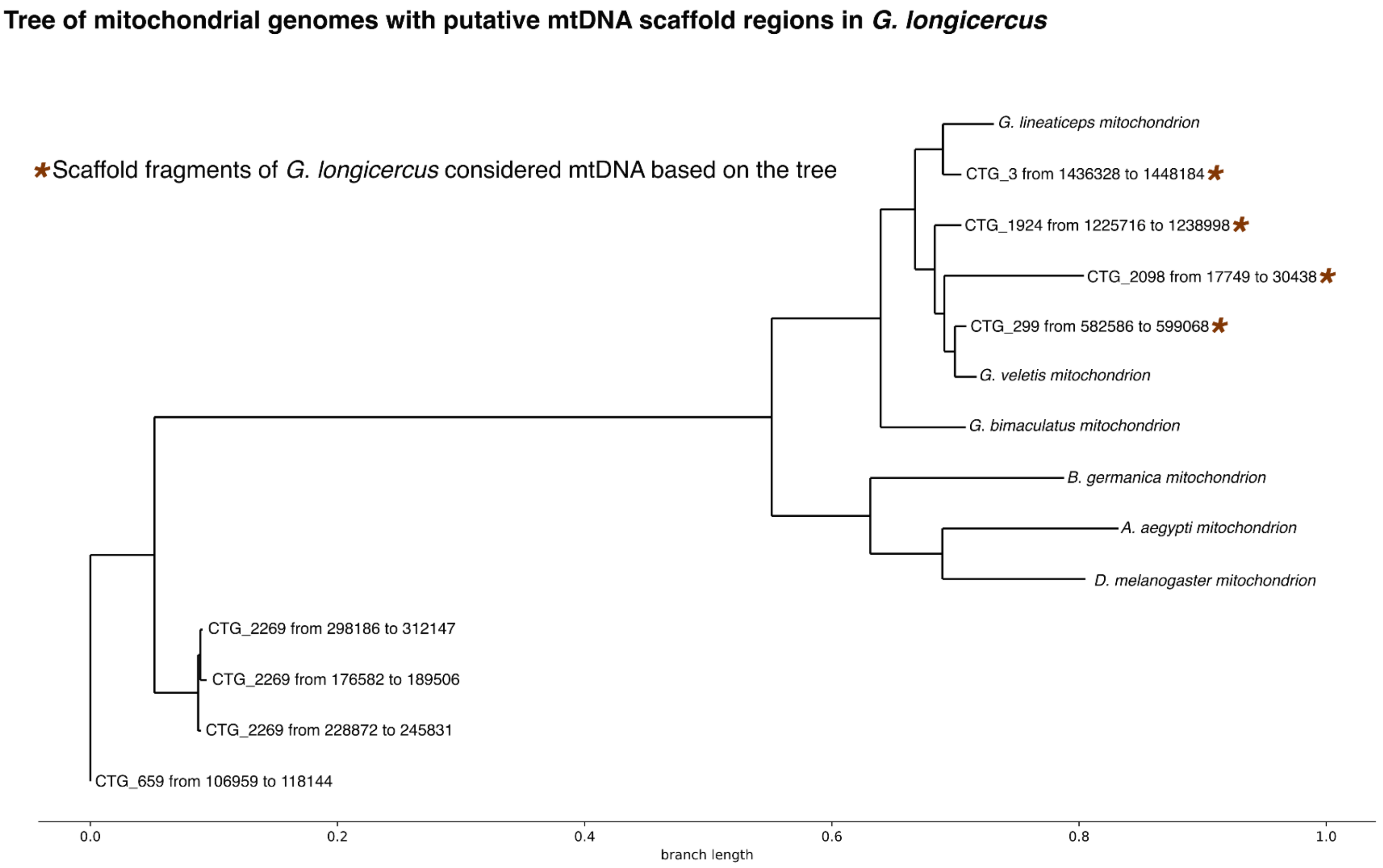

Supplementary Table S1: Masking report generated with RepeatMasker using RepBase library (v.20181026).

```
==================================================
file name: Glon_curated_04.06_genome.fasta
sequences:          1571
total length: 1850503451 bp  (1850503451 bp excl N/X-runs)
GC level:         40.32 %
bases masked:  193324530 bp ( 10.45 %)
==================================================
               number of      length   percentage
               elements*    occupied  of sequence
--------------------------------------------------
Retroelements            0          0 bp    0.00 %
   SINEs:                0          0 bp    0.00 %
   Penelope              0          0 bp    0.00 %
   LINEs:                0          0 bp    0.00 %
    CRE/SLACS            0          0 bp    0.00 %
     L2/CR1/Rex          0          0 bp    0.00 %
     R1/LOA/Jockey       0          0 bp    0.00 %
     R2/R4/NeSL          0          0 bp    0.00 %
     RTE/Bov-B           0          0 bp    0.00 %
     L1/CIN4             0          0 bp    0.00 %
   LTR elements:         0          0 bp    0.00 %
     BEL/Pao             0          0 bp    0.00 %
     Ty1/Copia           0          0 bp    0.00 %
     Gypsy/DIRS1         0          0 bp    0.00 %
       Retroviral        0          0 bp    0.00 %

DNA transposons          0          0 bp    0.00 %
   hobo-Activator        0          0 bp    0.00 %
   Tc1-IS630-Pogo        0          0 bp    0.00 %
   En-Spm                0          0 bp    0.00 %
   MULE-MuDR             0          0 bp    0.00 %
   PiggyBac              0          0 bp    0.00 %
   Tourist/Harbinger     0          0 bp    0.00 %
   Other (Mirage,        0          0 bp    0.00 %
    P-element, Transib)

Rolling-circles          0          0 bp    0.00 %

Unclassified:       1165452    126964360 bp    6.86 %

Total interspersed repeats:   126964360 bp    6.86 %


Small RNA:               0          0 bp    0.00 %

Satellites:              0          0 bp    0.00 %
Simple repeats:     1067779     66360170 bp    3.59 %
Low complexity:          0          0 bp    0.00 %
==================================================
* most repeats fragmented by insertions or deletions
  have been counted as one element

RepeatMasker version 4.1.4 , default mode
run with rmblastn version 2.14.1+
The query was compared to unclassified sequences in ".../RB.lib.fa"
FamDB:
```

Supplementary Table S2: Masking report generated with RepeatMasker using the *Gryllus bimaculatus* repeat library.

```
==================================================
file name: Glon_curated_04.06_genome.fasta
sequences:          1571
total length: 1850503451 bp  (1850503451 bp excl N/X-runs)
GC level:         40.32 %
bases masked:  946141920 bp ( 51.13 %)
==================================================
               number of      length   percentage
               elements*    occupied  of sequence
--------------------------------------------------
Retroelements       1579247    317705349 bp   17.17 %
   SINEs:           242628     30683556 bp    1.66 %
   Penelope          55506      8998650 bp    0.49 %
   LINEs:           920616    209727170 bp   11.33 %
    CRE/SLACS          427        32457 bp    0.00 %
     L2/CR1/Rex     270999     54621502 bp    2.95 %
     R1/LOA/Jockey  213492     48118959 bp    2.60 %
     R2/R4/NeSL      84676     18097736 bp    0.98 %
     RTE/Bov-B      108264     25243377 bp    1.36 %
     L1/CIN4         10294      2118428 bp    0.11 %
   LTR elements:    416003     77294623 bp    4.18 %
     BEL/Pao         20366      6948893 bp    0.38 %
     Ty1/Copia       35758      8138095 bp    0.44 %
     Gypsy/DIRS1    331419     59115452 bp    3.19 %
       Retroviral    25721      2761783 bp    0.15 %

DNA transposons     1352473    232178777 bp   12.55 %
   hobo-Activator   224978     38019411 bp    2.05 %
   Tc1-IS630-Pogo   395578     79041124 bp    4.27 %
   En-Spm                0            0 bp    0.00 %
   MULE-MuDR         29834      3651820 bp    0.20 %
   PiggyBac          14848      3783175 bp    0.20 %
   Tourist/Harbinger 15173      3494301 bp    0.19 %
   Other (Mirage,     6492      1497937 bp    0.08 %
    P-element, Transib)

Rolling-circles     146948     26870854 bp    1.45 %

Unclassified:       1239585    237575003 bp   12.84 %

Total interspersed repeats:   787459129 bp   42.55 %


Small RNA:           28722      3692528 bp    0.20 %

Satellites:          76478     10697846 bp    0.58 %
Simple repeats:     1122764    115385635 bp    6.24 %
Low complexity:      81721      5498068 bp    0.30 %
==================================================
* most repeats fragmented by insertions or deletions
 have been counted as one element

RepeatMasker version 4.1.4 , default mode
run with rmblastn version 2.14.1+
The query was compared to classified sequences in
".../Gbi_Rep_CombinedLibrary.lib.minlen50.nr.classified.filtered.fa"
FamDB:
```
